# Quantitative MRI in leukodystrophies

**DOI:** 10.1016/j.nicl.2023.103427

**Published:** 2023-05-03

**Authors:** Menno D. Stellingwerff, Petra J.W. Pouwels, Stefan D. Roosendaal, Frederik Barkhof, Marjo S. van der Knaap

**Affiliations:** a Amsterdam UMC Location Vrije Universiteit Amsterdam, Child Neurology, Emma Children’s Hospital, and Amsterdam Neuroscience, De Boelelaan 1117, Amsterdam, the Netherlands; b Amsterdam UMC Location Vrije Universiteit Amsterdam, Department of Radiology and Nuclear Medicine, and Amsterdam Neuroscience, De Boelelaan 1117, Amsterdam, the Netherlands; c Amsterdam UMC Location University of Amsterdam, Department of Radiology, Meibergdreef 9, Amsterdam, the Netherlands; d University College London, Institutes of Neurology and Healthcare Engineering, London, UK; e Vrije Universiteit Amsterdam, Department of Integrative Neurophysiology, Center for Neurogenomics and Cognitive Research, De Boelelaan 1105, Amsterdam, the Netherlands

**Keywords:** Quantitative MRI, White matter disorders, Leukodystrophies, Myelin imaging

## Abstract

Leukodystrophies constitute a large and heterogeneous group of genetic diseases primarily affecting the white matter of the central nervous system. Different disorders target different white matter structural components. Leukodystrophies are most often progressive and fatal. In recent years, novel therapies are emerging and for an increasing number of leukodystrophies trials are being developed. Objective and quantitative metrics are needed to serve as outcome measures in trials. Quantitative MRI yields information on microstructural properties, such as myelin or axonal content and condition, and on the chemical composition of white matter, in a noninvasive fashion. By providing information on white matter microstructural involvement, quantitative MRI may contribute to the evaluation and monitoring of leukodystrophies. Many distinct MR techniques are available at different stages of development. While some are already clinically applicable, others are less far developed and have only or mainly been applied in healthy subjects. In this review, we explore the background, current status, potential and challenges of available quantitative MR techniques in the context of leukodystrophies.

## Highlights

• Different quantitative MRI techniques offer complementary information on normal and abnormal white matter microstructure.
• Of the techniques that offer microstructural information, multi-shell DWI and myelin water imaging are particularly promising in leukodystrophies.
• Quantitative MRI may provide secondary outcome measures in leukodystrophy trials.
• Interpretation of modelled MRI measures in terms of pathology should take into account that models are based on normal white matter and may need to be adapted for abnormal white matter.
• Correlations between different MRI measures and disease severity should be further examined.

## 1 Introduction

Leukodystrophies constitute a large and heterogeneous group of genetic diseases targeting the white matter of the central nervous system (CNS). Conventional MRI, or qualitative structural MRI, provides images with anatomical details and includes T1- and T2-weighted imaging and fluid attenuated inversion recovery (FLAIR) imaging. Conventional MRI has a high diagnostic specificity and plays an important role in distinguishing individual leukodystrophies by pattern recognition, in visual rating scales and in volumetric analyses (Loes et al., 1994, Stellingwerff et al., 2021, van der Knaap et al., 2019). Because it depicts normal myelination, conventional MRI is also central in diagnosing delayed myelination (Wolf et al., 2020). Based on the degree and distribution of white matter signal abnormalities on T1- and T2-weighted images, hypomyelination can be distinguished from other pathologies (Wolf et al., 2020). FLAIR is helpful in detecting white matter rarefaction and cystic degeneration. Unfortunately, conventional MRI otherwise has a low specificity regarding underlying pathology. Different histopathological characteristics may underlie prominent white matter signal abnormalities, including demyelination, myelin vacuolization, other forms of white matter edema, and gliosis (van der Voorn et al., 2006). Quantitative MRI techniques could be of advantage by providing more specific and quantitative insight into different pathologies at tissue level. Several recent papers have reviewed quantitative MRI techniques that extract microstructural information (Alexander et al., 2019, Lee et al., 2021, Piredda et al., 2020, van der Weijden et al., 2022). Some of these techniques have been utilized to investigate normal white matter development (Dean et al., 2014, Deoni et al., 2012, Horska et al., 2002, Jelescu et al., 2015, Lebel and Deoni, 2018, Snook et al., 2005, van Buchem et al., 2001), while others have been applied in a wide range of brain diseases, such as stroke (Hui, 2022), neoplasms (Masjoodi et al., 2018, Nilsson et al., 2018), Alzheimer disease (Fu et al., 2020, Graff et al., 2022), and multiple sclerosis (MS) (Alotaibi et al., 2021, Filippi et al., 2017, Granziera, C., Wuerfel, J., Barkhof, F., Calabrese, M., De Stefano, N., Enzinger, C., Evangelou, N., Filippi, M., Geurts, J.J.G., Reich, D.S., Rocca, M.A., Ropele, S., Rovira, A., Sati, P., Toosy, A.T., Vrenken, H., Gandini Wheeler-Kingshott, C.A.M., Kappos, L., Group, M.S., 2021, Hagiwara et al., 2019).

Quantitative MRI techniques may also play a crucial role in monitoring disease progression. Multiple novel therapeutic approaches for leukodystrophies are in different stages of development and more are anticipated (van der Knaap et al., 2019), necessitating reliable outcome measures. These measures need to be robust, quantitative, reproducible, and sensitive to change. Preferably, changes in the measure are proportional to both changes in tissue microstructure and clinical parameters. In the context of multicenter studies, the results should ideally be site-independent.

In this review, we give an overview of the current knowledge of quantitative MRI techniques, with a focus on their potential ability to reflect white matter tissue changes in leukodystrophies.

## 2 White matter composition

The white matter contains axons, which conduct action potentials serving communication between neurons. Axons are cylinders with an intra-axonal diameter of 0.5–2 µm and lengths up to the extent of the entire spinal cord. Axons are mostly organized in bundles running in the same direction, but can also be fanning, kissing or crossing, depending on the specific region of the CNS (Lee et al., 2019).

Myelin sheaths are formed and maintained by a specific type of macroglial cells, the oligodendrocytes. Myelin is a modified extension of the oligodendrocyte cell membrane, wrapped around axons as a compacted multilayered sheath. The inner, cytoplasmic surfaces of the original cell membranes fuse, while the outer surfaces are closely apposed, seen as alternating electron-dense (“major dense lines”) and double less electron-dense (“intraperiod lines”) lines on electron microscopy. Myelin is very rich in lipids and contains specific protein and sugar moieties. Proteolipid protein and myelin basic protein are critical to ensure the proper structure, which is unique for myelin and not shared by any other membrane. Myelin is laid down in segments with uncovered areas (nodes of Ranvier) in between, allowing saltatory nerve conduction. Apart from guaranteeing efficient impulse conduction, myelin also insulates axons for structural and metabolic support (Stadelmann et al., 2019).

Astrocytes constitute another type of macroglia. They are involved in a wide array of functions: maintenance of ionic homeostasis, formation of the blood brain barrier, metabolic support to neurons and response to brain damage. Microglia are the scavenger cells or immune cells of the CNS (Vainchtein and Molofsky, 2020). Blood vessels transport blood to and from the CNS. They are separated from the CNS by the blood–brain barrier (Zarekiani et al., 2021).

## 3 Neuropathology in leukodystrophies

The pathogenesis of leukodystrophies may involve defects in all white matter components. Based on cellular pathology, leukodystrophies can be classified into the following categories: myelin disorders, astrocytopathies, leuko-axonopathies, microgliopathies, leuko-vasculopathies, and a category of unknown cellular pathology. Myelin disorders are further divided into disorders with hypomyelination, demyelination and myelin vacuolization. The underlying pathological processes are typically complex and leukodystrophies may belong to multiple categories (van der Knaap and Bugiani, 2017).

**Fig. 1:**
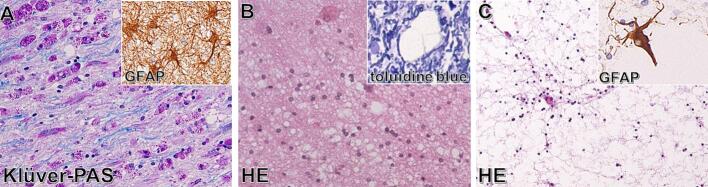
Heterogeneous neuropathology of leukodystrophies. A) Metachromatic leukodystrophy (MLD) is characterized by loss of myelin (Klüver-periodic acid-Schiff (PAS), blue) and accumulation of myelin debris in macrophages (pink). Dense astrogliosis is present (inset in A, GFAP staining). B) Megalencephalic Leukodystrophy with subcortical Cysts is characterized by white matter vacuolization (haematoxylin and eosin (HE)) without lack of myelin (inset in B, toluidine blue). C) Vanishing White Matter is characterized by loss of virtually all white matter components (HE) and a profound lack of astrogliosis (inset in C, GFAP staining). Magnification of all staining is 400x.\

Adapted from van der Knaap and Bugiani, 2018

A wide array of neuropathological changes can occur in leukodystrophies, depending on the specific type of leukodystrophy, its severity and the disease stage. Fig. 1 shows histopathological stainings of three distinct leukodystrophies, exemplary of the heterogeneous neuropathology. Changes may be primary, directly related to the etiology of the disease, or secondary to other pathological changes. The amount, thickness, compactness or chemical composition of myelin sheaths may be abnormal. In many leukodystrophies, axonal loss occurs secondary to myelin loss, but axonal degeneration and loss may also be primary. The axonal diameter may change. White matter damage is usually accompanied by astrogliosis, but a disproportionate lack of astrogliosis may be observed in leukodystrophies characterized by suboptimal repair potential. The cell density in the white matter may change, for instance as a result of increased numbers of astrocytes, oligodendrocyte precursors or invasion by white blood cells (van der Knaap and Bugiani, 2017). Various types of white matter edema can occur, such as vasogenic, interstitial, intramyelinic and cytotoxic edema (Patay, 2005). In intramyelinic edema, the size of the vacuoles is highly variable.

## 4 Quantitative MRI

### 4.1 Search strategy and overview of quantitative MRI techniques

A literature search with the search terms “MRI AND leukodystrophy” was performed on PubMed in April 2023, resulting in 1196 publications. We selected articles that used quantitative MRI methods to study leukodystrophies, and excluded non-human studies and non-brain studies. The number of papers thus selected for further analysis was 62. We also search the literature for reviews and key references on the different quantitative MRI techniques potentially relevant for leukodystrophies.

Each quantitative MRI technique is based on a specific use of magnetic field gradients and radio-frequent pulses, comprised in the pulse sequence, to produce data with specific characteristics. Quantitative MRI techniques may provide model-free and model-based MRI measures. Model-free measures provide a representation of the derived MR signal in terms of voxel parameters such as proton density, relaxation times, or diffusion coefficients (Table 1). Model-based measures, sometimes named in-vivo MRI histology, aim to describe microstructural components by fitting the MR signal within a voxel to a biophysical model (Table 2) (Novikov et al., 2019, Weiskopf et al., 2021).

**Table 1:** Quantitative MR techniques (non-exhaustive).

Techniques	Measures
Ultrashort echo time (UTE)double-echo sliding inversion recovery (DESIRE)	Density of short T2 component
Magnetization transfer (MT) imaging	MT ratio, quantitative MT
Inhomogeneous magnetization transfer (ihMT)	ihMT ratio
Quantitative susceptibility mapping (QSM)	Magnetic susceptibility
Multi-compartment relaxometry (MCR)	PD, T1, T2, T2* relaxation times
Multi-echo spin echo (MESE)	
Gradient and spin echo (GRASE)	
Multi-echo T2 relaxation imaging with compressed sensing (METRICS)	
Multi-echo gradient echo (MGRE)	
Multicomponent driven equilibrium steady-state observation of T1 and T2 (mcDESPOT)	
Quantification of relaxation times and proton density by multi-echo acquisition of a saturation-recovery using turbo spin-echo readout (QRAPMASTER) FAST-T2	
Short-TR adiabatic inversion-recovery (STAIR)	Myelin water signal
Magnetic resonance fingerprinting (MRF)	PD, T1, T2, T2* relaxation times
Diffusion-weighted imaging (DWI) Diffusion tensor imaging (DTI) Diffusion kurtosis imaging (DKI)	Mean diffusivity, radial diffusivity, axonal diffusivity, fractional anisotropy Mean kurtosis, radial kurtosis, axonal kurtosis
MR spectroscopy (MRS)	Metabolite (NAA, Cre, Cho, Lac, etc,) concentrations and ratio’s

**Table 2:** Model-based quantitative MRI (non-exhaustive).

Model	Estimates	Sequence
Myelin water imaging (MWI)	Myelin water fraction	Any MCR-sequence, STAIR
Neurite orientation dispersion and density imaging (NODDI)	Neurite density, neurite orientation dispersion, free water fraction	Multi-shell diffusion
SyMRI	Myelin and edema maps	QRAPMASTER

In this review, we discuss quantitative techniques that are potentially relevant for leukodystrophies. For each method, we shortly describe the MR principles, the findings in healthy subjects and, if available, in patients with leukodystrophies or other white matter disorders, and the future directions. Without suggesting a chronological or hierarchical order, we start with discussing relaxation-based techniques (4.2 multi-compartment relaxometry, 4.3 STAIR, 4.4 SyMRI, 4.5 MR fingerprinting), continue with diffusion-based techniques (4.6 diffusion weighted imaging) and methods based on the macromolecular content of the brain (4.7 ultrashort echo time imaging, 4.8 magnetization transfer imaging), followed by susceptibility-based (4.9 QSM) techniques. We finish section 4 with MR spectroscopy (4.10), which is based on brain metabolites.

### 4.2 Multi-compartment relaxometry

Myelin is a major white matter component and MRI techniques providing variables sensitive to myelin content or properties are highly desirable. The differences in relaxation times T2, T2* and T1 of water residing in different compartments enable an indirect estimation of myelin content, and can be performed using multi-compartment relaxometry. For example, at clinical field strengths up to 3 T, T2-relaxation times of myelin water, i.e. water within and between myelin sheaths, typically are under 40 ms, whereas T2 of intra- and extracellular water is between 70 and 95 ms (MacKay et al., 1994) and T2 of CSF is over 1 s. A commonly derived measure is the myelin-water fraction (MWF), calculated by the myelin water signal divided by the total water signal. Various imaging protocols exist to perform multi-compartment T2 relaxometry by fitting a multi-exponential equation to the signal decay curve as function of TE.

The Carr-Purcell-Meiboom-Gill (CPMG) sequence, a multi-echo spin-echo sequence (MESE), was the first multi-compartment T2 relaxometry technique, and is often considered the gold standard. The acquisition time is long, with a single slice initially taking 26 min (MacKay et al., 1994). A sequence that combines gradient and spin echoes (GRASE) has been developed to shorten the acquisition time, while still producing predominantly T2 contrast. With 2D- and 3D-GRASE sequences, whole-brain MWF-maps can be obtained within 7.5 and 15 min, respectively (Drenthen et al., 2019, Prasloski et al., 2012). A disadvantage of GRASE sequences is unwanted smoothing due to faster T2* decay and thereby lower SNR in the periphery of k-space (Dvorak et al., 2020). By using compressed sensing, 3D multi-echo T2 relaxation imaging with compressed sensing (METRICS) aims to overcome the problems associated with GRASE, while maintaining high SNR and acceptable acquisition times (7.5–10 min) (Dvorak et al., 2020). With the FAST-T2 sequence, the T2 weighting is implemented prior to the readout phase, by applying so-called T2 prep modules. FAST-T2 allows acquisition of a whole brain volume with 5 mm slice thickness within 4 min, by reducing the number of TEs and using multi-slice spiral readout (Nguyen et al., 2016). The MWF values derived with GRASE, METRICS and FAST-T2 are similar to MWF-values derived from a CPMG sequence (Drenthen et al., 2019, Dvorak et al., 2020, Nguyen et al., 2016, Prasloski et al., 2012).

Multi-compartment T2* relaxometry can be performed with a multi-echo gradient echo (MGRE) sequence, which is faster than T2 relaxometry, because a train of gradient readouts is faster than spin echoes (Du et al., 2007). A recent study combined a MGRE sequence with variable flip angles, resulting in a method which corrects for T1-dependency (Chan and Marques, 2020). The mcDESPOT technique generates whole-brain T1-, T2- and MWF maps within 15 min (Deoni et al., 2008). McDESPOT-derived MWF values are significantly higher than CPMG-derived MWF values (Zhang et al., 2015)(Fig. 4), and strongly depend on acquisition schemes and post-processing algorithm (West et al 2019). For in-depth technical reviews on myelin water imaging, we refer to the review papers by Lee (Lee et al., 2021) and Piredda (Piredda et al., 2020).

**Fig. 2:**
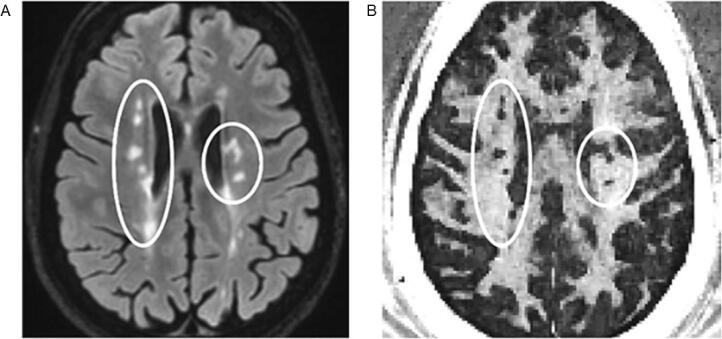
A) T2-weighted FLAIR and B) ultrashort TE subtraction images in a 45-year-old woman with MS, indicating a lower signal intensity in the UTE image in lesions that appear hyperintense on FLAIR. Adapted with permission from Ma et al., 2020.

**Fig. 3:**
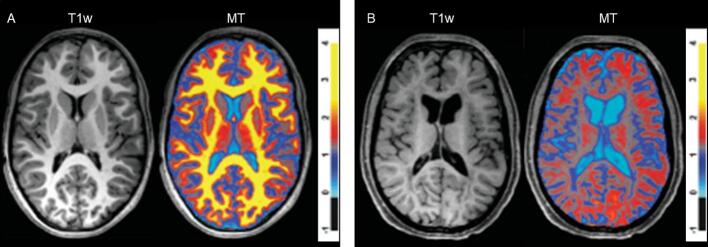
A) Normal magnetization transfer (MT) findings in an 11-year-old healthy control. B) Decreased MT ratios are visible in the cerebral white matter of a 13-year-old subject with the leukodystrophy Hypomyelination and Atrophy of Basal ganglia and Cerebellum, while the T1-weighted image still shows a relatively normal hyperintense T1-signal. Adapted with permission from Dreha-Kulaczewski et al., 2012.

**Fig. 4:**
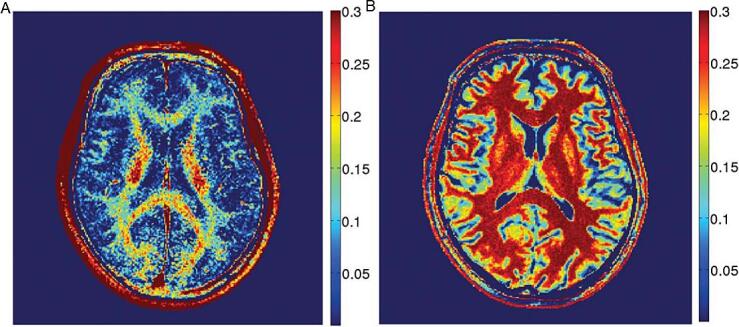
Myelin water fraction maps from a healthy subject with GRASE (A) and mcDESPOT (B). The two maps are shown with the same color bar (with values ranging from 0 to 0.3). The maps show higher MWF in white matter than in gray matter, but values differ between both techniques. Adapted with permission from Zhang et al., 2015.

**Fig. 5:**
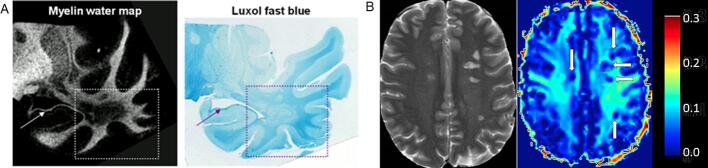
A) Postmortem myelin water map and corresponding Luxol fast blue-staining of the temporal lobe region of a multiple sclerosis (MS) patient. B) T2-weighted image and corresponding multi-echo spin echo (MESE) sequence derived myelin water fraction-map of an MS patient, showing a reduced myelin water fraction in the MS lesions (arrows). A) Adapted with permission from Laule et al., 2008; B)

Adapted from Faizy et al., 2016

Most studies on multi-compartment relaxometry concern normal development, providing typical trajectories of MWF and normative data in various age groups (Deoni et al., 2012, Geeraert et al., 2018, Morris et al., 2020). The trajectory of MWF does not exactly follow the T1 and T2 trajectories, and depends on the investigated region of interest (ROI), at least partly due to differences in underlying microstructure (Groeschel et al., 2016). The correlation between MWF and histopathology-derived myelin content was strong in MS patients (Faizy et al., 2016, Laule et al., 2008) (Fig. 5). In a cohort of primary progressive MS patients, MWF values were significantly correlated with clinical disability (Kolind et al., 2012). In a patient with Krabbe disease, the MWF was evidently lower than in control subjects, but stable over time after hematopoietic stem cell transplantation (Laule et al., 2018). In patients with metachromatic leukodystrophy (MLD), MWF was also evidently lower than in controls (Martin et al., 2020).

It is important to realize that a priori constraints of the model may prevent visualization of water compartments with other relaxation characteristics. Ideally, the full spectrum of relaxation times should be considered. For example, studies in subjects with MS and phenylketonuria showed additional water compartments with T2 relaxation times between 200 and 800 ms in a subset of MS lesions and normal-appearing white matter, and also in white matter T2-hyperintensities in subjects with phenylketonuria (Laule et al., 2007a, Laule et al., 2007b). This additional signal may originate from, for instance, intramyelinic vacuoles or an increased amount of extracellular water (Laule et al., 2007b). The T2 relaxation time of myelin water is also influenced by myelin sheaths being thinner than normal, less compact or having an altered chemical composition (Odrobina et al., 2005). Consequently, in abnormal tissue, relaxation times of water compartments may be changed, and what is measured as “MWF” cannot be regarded straightforwardly as being representative of the myelin content.

### 4.3 Direct myelin water imaging

The short-TR adiabatic inversion-recovery (STAIR) method directly derives the myelin water signal, and is therefore not a multi-compartment relaxometry method. First, water components with a longer T2 and T1, for instance from intra- and extracellular water, are suppressed and a signal is obtained that mainly originates from myelin water, avoiding the need for modelling. If this myelin water image is divided by a proton density image, an apparent MWF can be calculated. The MWF values derived with STAIR are similar to MWF-values derived from a CPMG sequence (Ma et al., 2022). The method has so far been applied in a small cohort of MS patients (Ma et al., 2022).

### 4.4 Synthetic MRI

Synthetic MRI is based on the synthesis of contrast-weighted images from measured relaxation times and/or proton density (Bobman et al., 1985). SyMRI is a proprietary technique using the principles of synthetic MRI (Hagiwara et al., 2017). The applied sequence in SyMRI is called QRAPMASTER, which simultaneously determines T1 and T2 relaxation times and proton density. QRAPMASTER relies on a saturation pulse, and after a varying delay time, a multi-echo spin-echo acquisition (Warntjes et al., 2008). The estimated relaxation times and proton density can be converted to FLAIR, T1-weighted and T2-weighted images (Warntjes et al., 2008). Myelin and edema maps can also be constructed, by fitting four water compartments with their own fixed or constrained proton density and relaxation properties to each voxel (Warntjes et al., 2016).

**Fig. 6:**
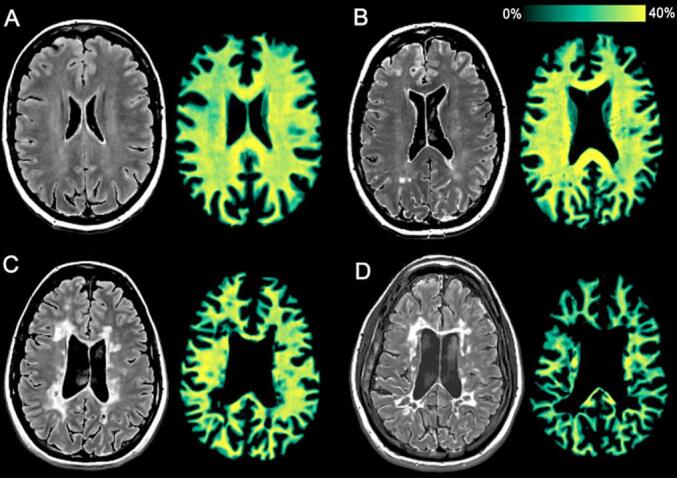
T2-weighted FLAIR and corresponding SyMRI myelin map in 4 subjects. A) A 56-year-old healthy control. B) A 53-year-old patient with primary progressive multiple sclerosis (MS). C) A 39-year-old patient with relapsing–remitting MS. D) A 40-year-old patient with secondary progressive MS. The myelin map shows the lowest values in the patient with secondary progressive MS, and the highest values in the healthy control. Adapted with permission from Ouellette et al., 2020.

A correlation between SyMRI myelin estimation and myelin staining was found in brain specimens (Warntjes et al., 2016). Also, correlations were found between MTR-derived myelin volume fractions and SyMRI myelin estimations in white matter of controls (Hagiwara et al., 2018). With SyMRI, a lower myelin volume fraction was found in MS patients than in controls (Fig. 6) (Ouellette et al., 2020, Warntjes et al., 2016). SyMRI metrics were similar in patients with Adult Leukoencephalopathy with Spheroids and Pigmented glia and patients with MS (Mangeat et al., 2020). Apart from a few case reports, the technique has not been applied in other white matter diseases.

SyMRI offers a range of quantitative information and can be implemented on clinical MR systems. A whole-brain 3D acquisition is clinically feasible, and even shorter acquisition times of 6 min can be obtained with compressed sensing (Fujita et al., 2021). Similar to mcDESPOT, SyMRI-derived MWFs are significantly higher than those found with CPMG (Warntjes et al., 2016), possibly due to the complexity of the models, and bias caused by using fixed values (Lankford and Does, 2013, Piredda et al., 2020, West et al., 2019).

### 4.5 Magnetic resonance fingerprinting

Magnetic resonance fingerprinting (MRF) also provides multiple tissue characteristics within a single acquisition (Ma et al., 2013, Panda et al., 2017). Quantitative T1- and T2-maps are normally acquired by changing only one acquisition parameter. MRF uses a pseudo-random acquisition scheme with variable TEs, flip angles, and TIs, which results in an under-sampled signal, the so-called fingerprint. This fingerprint is matched with a simulated fingerprint within a dictionary, which describes voxels with all kinds of tissue properties. The resulting quantitative maps have been shown to be accurate and repeatable in healthy controls (Buonincontri et al., 2021, Korzdorfer et al., 2019, Panda et al., 2017).

Using MRF to measure MWFs may be useful in leukodystrophies, but has not been described in leukodystrophies or other white matter disorders yet. MRF was applied in the Baby Connectome Project and showed clear differences in MWF between subjects ranging from 0 to 5 years of age (Chen et al., 2019). Surprisingly, even in the corpus callosum, the MWF was negligibly small up to 6 months (Chen et al., 2019), whereas on conventional imaging, myelination of the corpus callosum can already be appreciated by then (Barkovich et al., 1988).

### 4.6 Diffusion-weighted MRI

While the previously described techniques tend to focus on estimation of myelin content, a broader scope is desired, as neuropathology in leukodystrophies is not restricted to myelin (van der Knaap and Bugiani, 2018). Diffusion-weighted MRI relies on Brownian motion of water, which can be probed using strong gradients. Voxels with restricted diffusion have a low value of the apparent diffusion coefficient (ADC), and can typically be recognized as high signal on diffusion-trace images. Diffusion-weighted MRI has been used extensively. For clinical sequences, using diffusion gradients with b-values around 1000 s/mm^2^ at field strengths up to 3 T, ADC values are quite robust, and reference values for specific tissues and ages are well known (Belli, G., Busoni, S., Ciccarone, A., Coniglio, A., Esposito, M., Giannelli, M., Mazzoni, L.N., Nocetti, L., Sghedoni, R., Tarducci, R., Zatelli, G., Anoja, R.A., Belmonte, G., Bertolino, N., Betti, M., Biagini, C., Ciarmatori, A., Cretti, F., Fabbri, E., Fedeli, L., Filice, S., Fulcheri, C.P., Gasperi, C., Mangili, P.A., Mazzocchi, S., Meliado, G., Morzenti, S., Noferini, L., Oberhofer, N., Orsingher, L., Paruccini, N., Princigalli, G., Quattrocchi, M., Rinaldi, A., Scelfo, D., Freixas, G.V., Tenori, L., Zucca, I., Luchinat, C., Gori, C., Gobbi, G., Italian Association of Physics in Medicine Working Group on, M.R.I., 2016, Morriss et al., 1999). With diffusion tensor imaging (DTI), not only the magnitude but also the direction of diffusion can be depicted, using a 3D ellipsoid with corresponding lengths (eigenvalues) and directions (eigenvectors). From the eigenvalues, metrics as fractional anisotropy (FA), axial diffusivity (AD), radial diffusivity (RD), and mean diffusivity (MD, equivalent to ADC) can be calculated. These metrics are particularly useful for characterizing white matter areas with coherent fiber bundles, and can be considered indicative of microstructural integrity, axonal integrity, degree of myelination, and membrane density, respectively in such areas (Alexander et al., 2007, Assaf and Pasternak, 2008, Lebel et al., 2008). The majority of white matter voxels, however, contains crossing fibers, in which interpretation of DTI metrics is less straightforward (Jones and Cercignani, 2010).

Although widely used, the tensor model has limitations because it assumes pure Gaussian diffusion (Jensen and Helpern, 2010). Multi-shell DWI, with non-diffusion weighted images (b0) and at least two higher b-values, allows estimation of non-Gaussian diffusion using diffusion kurtosis imaging (DKI) (Jensen and Helpern, 2010). Additionally, it facilitates the description of tissue properties with biophysical models. A model like neurite orientation dispersion and density imaging (NODDI) can estimate orientation dispersion index (ODI), neurite density index (NDI) and the free water fraction (Zhang et al., 2012). The intra-neurite compartment is modelled as restricted non-Gaussian diffusion, the extra-neurite compartment as hindered Gaussian diffusion, and the CSF compartment as isotropic Gaussian diffusion with a fixed diffusivity (Zhang et al., 2012). NDI is more homogeneous throughout the white matter than FA, as NDI is less affected by the fiber configuration (e.g. fanning or crossing fibers). With single-shell data, the estimation of NDI is unreliable (Timmers et al., 2016, Zhang et al., 2012). A biophysical model like NODDI aims to reflect tissue microstructure, but it remains a statistical approximation of the diffusion signal of a (large) voxel. The model assumes simplified shapes and the anatomical detail and complexity of histology is not reached (Alexander et al., 2019). An alternative to voxel-based analysis, is to compare individual populations of fibers within a specific voxel, also named fixel-based analysis. With fixel-based analysis it is possible to specifically quantify the density or cross-section of a subset of fibers within a voxel and to perform more complex fiber-specific statistics (Dhollander et al., 2021). For in-depth reviews of brain microstructure and biophysical models in DWI, we refer to Alexander (Alexander et al., 2019) and Jelescu (Jelescu et al., 2020).

DWI may show discriminative features for different leukodystrophies. Vasogenic and interstitial edema leads to facilitated diffusion (i.e. higher diffusivity values). Cytotoxic edema leads to restricted diffusion, whereas diffusion in intramyelinic edema depends on the size of the vacuoles (Patay, 2005). Facilitated diffusion is most frequently seen in leukodystrophies due to loss of white matter tissue and increased water content (van der Voorn et al., 2006). In several leukodystrophies restricted diffusion may also be observed. For instance, in Leukoencephalopathy with Brainstem and Spinal cord involvement and Lactate elevation and ClC-2 chloride channel deficiency, restricted diffusion is present in part of the abnormal white matter, representing intramyelinic micro-vacuolization (Depienne et al., 2013, Steenweg et al., 2016, Yamashita et al., 2013). In Megalencephalic Leukoencephalopathy with subcortical Cysts the intramyelinic vacuoles are large and cause facilitated diffusion (van der Voorn et al., 2006). In Vanishing White Matter, increased cellularity in relatively spared regions leads to restricted diffusion, whereas rarefied or cystic regions have facilitated diffusion (van der Lei et al., 2012). In MLD, sulfatide accumulation in distended perivascular macrophages may result in white matter stripes with restricted diffusion (Oguz et al., 2004, Seidl et al., 2008, Sener, 2003, van Rappard et al., 2018b).

DTI-metrics change with normal myelination and differ per brain area. FA increases and MD, RD, and AD decrease with increasing myelin content during normal development (Feng et al., 2019, Lebel et al., 2008). FA is decreased in both normal-appearing white matter and lesions of MS patients, indicating its sensitivity for pathology. Because a decrease in FA occurs in almost all types of pathology, it is unsuitable for distinguishing the underlying pathological change; however, the value itself contains information about the severity of white matter damage (Assaf and Pasternak, 2008, Filippi et al., 2017). Changes in RD and AD may be more indicative of myelin pathology and axonal pathology, respectively (Aung et al., 2013). Indeed, in MS increases in AD are typically smaller than increases in RD, in line with mainly myelin pathology (Roosendaal et al., 2009).

DTI-results have been reported for several leukodystrophies. Compared to controls, patients with hypomyelination have lower white matter FA-values (Steenweg et al., 2016). The lower value of FA is mainly due to a higher value of RD, whereas the difference in AD is smaller, likely due to the normal axonal density in hypomyelination (Steenweg et al., 2016). Leukodystrophies with demyelination, myelin macro-vacuolization, or cystic white matter degeneration have lower FA-values than patients with hypomyelination (van der Voorn et al., 2006). DTI has been used quite extensively in Krabbe research and provides a sensitive biomarker (Escolar et al., 2009, Guo et al., 2001, Gupta et al., 2015, Ito et al., 2001, Poretti et al., 2014, Poretti et al., 2016, Provenzale et al., 2005). Presymptomatic patients may already have DTI-abnormalities, as shown by a (slighty) reduced FA in the corticospinal tract in neonates with Krabbe disease (Escolar et al., 2009). In Krabbe disease these metrics were also predictive for functional outcome after transplantation (Gupta et al., 2015). In MLD, the AD can be reduced in perivascular areas due to sulfatide accumulation in macrophages, but may increase at a later stage due to loss of both myelin and axons (van Rappard et al., 2018b). DTI-measures are sensitive biomarkers and have previously shown their value in various other leukodystrophies, such as Canavan disease (Janson et al., 2006, Kimiskidis et al., 2017), Adult-onset autosomal Dominant Leukodystrophy (Zanigni et al., 2015), hypomyelinating PLP1-related disorders (Sarret et al., 2018).

**Fig. 7:**
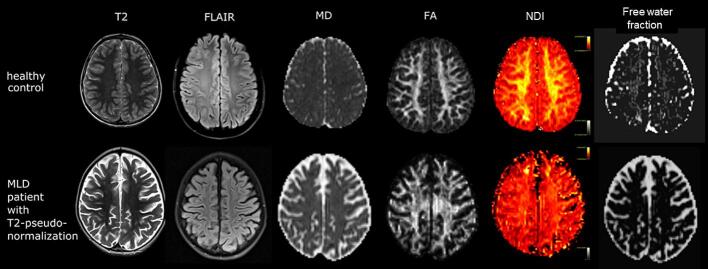
Healthy control (upper row) and a patient with metachromatic leukodystrophy (MLD, lower row) with normal T2-weighted signal intensity in cerebral white matter, which was T2-hyperintense in previously obtained MR scans. Besides DTI-parameters such as MD and FA, NODDI can model the neurite density index (NDI) and the free water fraction. The neurite density is clearly reduced in the normal looking white matter in the MLD patient, confirming pseudo-normalization.

Adapted from Martin et al., 2020

NODDI may be more specific to microstructural properties and has been widely applied in MS. Both lesions and normal-appearing white matter have lower NDI values than healthy white matter, indicating the sensitivity of the method (Collorone et al., 2019, Granberg et al., 2017). In a post-mortem study, NODDI proved useful in characterizing the pathology of demyelinated lesions in spinal cord of MS patients, as the ODI was increased and matched the histology-derived orientation dispersion measure. DTI measures were less specific to the described histological changes (Grussu et al., 2017). In patients with galactosemia, NODDI showed extensive white matter abnormalities with decreased NDI and increased ODI. NDI and ODI maps contained more voxels with abnormal values than the FA map (Timmers et al., 2015). NODDI has also been applied in a few patients with MLD to investigate white matter regions that had a normal signal intensity of T2-weighted images, but were T2-hyperintense in previously obtained MR scans. This pseudonormalization was hypothesized to be caused by increasing accumulation of non-degradable lipids in macrophages. As NDI and the free water fraction were decreased, and ODI was increased compared to controls, NODDI confirmed that the tissue was not normal (Fig. 7) (Martin et al., 2020).

### 4.7 Ultrashort echo time imaging

Direct imaging of myelin would be the most straightforward technique to quantify myelin. Protons within myelin lipid, protein, and sugar macromolecules have very short T2-relaxation times, typically less than 1 ms, and can therefore only be detected with ultrashort echo times (UTEs) (Ma et al., 2020, Sheth et al., 2016). A so-called myelin image can be obtained by subtracting an image with short TE (in ms range) from an image with a UTE (in μs range). Additional water suppression is necessary, because water protons are much more abundant than myelin protons (Sheth et al., 2016). Due to variation in T1-relaxation times, water suppression is suboptimal on standard inversion recovery sequences. A 3D UTE sequence named DESIRE uses several inversion times (TI) to optimize water suppression, enabling whole-brain imaging within 10 min (Ma et al., 2020).

UTE sequences are technically challenging due to hardware limitations of clinical MR systems and mediocre signal-to-noise ratio (SNR) (Seifert et al., 2017). The technique has not been applied widely. UTE imaging has been performed in MS brain specimens, in patients with MS, and in healthy volunteers. The signal intensity in MS lesions was significantly lower than in normal-appearing white matter, consistent with myelin loss in the lesions (Fig. 2) (Ma et al., 2020). Also, the signal intensity in normal-appearing white matter was lower in MS patients than in healthy controls (Jang et al., 2021). Whether the UTE signal intensities are proportional to actual myelin content still has to be established. The very short T2 is shared by lipid, protein, and sugar molecules in other membranes. Myelin constitutes approximately 50% of normal white matter and myelin density is typically less in abnormal white matter (van der Knaap and Bugiani, 2017). As such, UTE imaging does not solely reflect myelin, but rather protons in membrane macromolecules.

### 4.8 Magnetization transfer imaging

Instead of direct imaging of macromolecules with UTE, magnetization transfer imaging exploits the transfer of magnetization between different proton pools to extract microstructural information. With an off-resonance radiofrequency pre-pulse, macromolecular protons are excited. Through interaction between macromolecular protons and water protons this magnetization is partly transferred to free water protons. This transfer leads to a partial saturation of the pool of free water protons, thereby reducing the MR signal. The magnetization transfer ratio (MTR) represents the signal loss between the image without pre-pulse (M_0_) and the image with magnetization pre-pulse (MT^+^) [1 - MT^+^ / M_0_] (van Buchem et al., 2001). The MTR depends on the macromolecular content of tissues and is therefore considered indicative of the amount of myelin and axonal membranes (Schmierer et al., 2007, van Buchem et al., 2001). The MTR is also influenced by the tissue water content (Schmierer et al., 2007).

Inhomogeneous MT (ihMT) has been developed in an effort to make MTR more specific to the myelin content (Duhamel et al., 2019, Varma et al., 2015). It is based on dipolar order relaxation, which is much longer in lipids and proteins in membranes than in small molecules, making the method sensitive to myelin (Varma et al., 2015). Further specificity for myelin might be obtained by focusing on specific dipolar relaxation times (Alsop et al., 2022). For an in-depth overview on magnetization transfer imaging, including quantitative MT, we refer to the review by Sled (Sled, 2018).

MT imaging has been applied widely in MS. Both in lesions and in normal-appearing white matter, MTR is lower than in white matter of controls, which is thought to be partly due to an increased water content (Moll et al., 2011). In a recent study comparing ihMT and MTR, ihMT correlated with MS disease severity, whereas MTR did not. Conversely, ihMT had a lower sensitivity to detect differences from control values in normal-appearing white matter. These observations indicate that ihMT may be less sensitive to changes in water content and reflect myelin content more reliably (Van Obberghen et al., 2018). Application of MT imaging in leukodystrophies showed decreased values of MTRs in white matter (Fig. 3) (Dreha-Kulaczewski et al., 2012, Martin et al., 2020, Steenweg et al., 2016, van der Voorn et al., 2006). Hypomyelination, demyelination and myelin vacuolization are pathologies with a similar drop in MTR relative to controls, while in fact the white matter water content is highly increased and myelin is not lost in myelin vacuolization (van der Knaap et al., 1996, van der Voorn et al., 2006). Cystic degeneration is associated with the strongest decrease in MTR (van der Voorn et al., 2006), reflecting the loss of all tissue structures, which are replaced by water. In X-linked adrenoleukodystrophy, MTR was able to show two lesional zones in 7 out of 8 patients with a cerebral lesion with contrast enhancement, corresponding to a zone with early demyelination and a zone with gliosis (Melhem et al., 1996).

The fact that MTR is not determined by a single tissue component but influenced by several tissue properties, in particular the content of myelin, other cellular and subcellular membranes and tissue water, makes interpretation of the results challenging. Sophisticated MT-based techniques like ihMT may provide results that are more specifically related to a tissue component and may thus better distinguish between pathologies, but have yet to be validated (Schmierer et al., 2007, Van Obberghen et al., 2018). Not only myelin but also other membranes consist of phospholipid bilayers, and ihMT therefore does not solely reflect myelin content (Alsop et al., 2022, Varma et al., 2015).

### 4.9 Quantitative susceptibility mapping

Quantitative susceptibility mapping (QSM) is based on susceptibility, the degree that a substance can be magnetized by an applied magnetic field (Schenck, 1996). Differences in susceptibility of tissue lead to variations in the local magnetic field, expressed in parts per million (ppm) (Harada et al., 2022, Schenck, 1996). Calcium, lipids, proteins and myelin are diamagnetic (lower susceptibility than water), whereas iron and deoxygenated blood are paramagnetic (higher susceptibility than water) (Hametner et al., 2018, Harada et al., 2022, Vinayagamani et al., 2021). QSM could therefore play a role in quantification of iron, calcium, microbleeds and myelin content. QSM uses data acquired with susceptibility-weighted imaging, which is often already part of a clinical scanning protocol. Major benefits are the short acquisition time and high spatial resolution. The interpretation of QSM results may benefit from an option to decompose the voxel-wise signal into sub-voxel paramagnetic and diamagnetic components (Chen et al., 2021). For a review on technical considerations and clinical applications on QSM, we refer to Vinayagamani (Vinayagamani et al., 2021).

In rodents, promising correlations were seen between QSM measures and myelin histopathology (Argyridis et al., 2014, Lodygensky et al., 2012). However, the sensitivity of QSM to myelin changes in humans is a topic of debate, as in human MS specimens no relation was found between QSM and myelin histopathology (Wiggermann et al., 2017). This might be due to higher iron content in humans (Argyridis et al., 2014). Susceptibility of white matter and gray matter are clearly different. In normal development and aging, temporal changes are mainly driven in the white matter by changes in myelin content, and in the deep gray matter structures by changes in iron content. Initially, white matter susceptibility decreases during childhood and adolescence due to diamagnetic properties of myelin, after which it increases with age due to myelin breakdown. The susceptibility changes over time differ per white matter region. In deep gray matter structures, susceptibility increases exponentially over time due to iron accumulation (Li et al., 2014, Zhang et al., 2019).

QSM has been used extensively in MS research to characterize lesions, for instance by illustrating the central vein sign and iron rings (Absinta et al., 2016, Dal-Bianco et al., 2017, Sati et al., 2016). QSM also enables staging of MS lesions due to temporal changes in iron and myelin content within MS lesions (Chen et al., 2014, Harada et al., 2022, Vinayagamani et al., 2021). Iron plays an important role in neuroinflammation (Ward et al., 2022), and as inflammatory processes may occur in leukodystrophies, QSM is an interesting MR technique to explore in leukodystrophies.

The available literature of QSM in leukodystrophies is limited. QSM may provide information on neuropathology in leukodystrophies, in which calcification, iron accumulation, or microbleeds occur. For instance, calcium deposits are frequently present in Leukoencephalopathy with Calcifications and Cysts, Aicardi–Goutières syndrome, Coates Plus syndrome, Adult-onset Leukoencephalopathy with axonal Spheroids and Pigmented glia, mitochondrial leukodystrophies and genetic vasculopathies (van der Knaap et al., 2019). One case report on Leukoencephalopathy with Calcifications and Cysts confirmed the ability of QSM to differentiate between calcifications and microbleeds (Raab et al., 2017).

### 4.10 MR spectroscopy

Among the quantitative MR methods currently used, proton MR spectroscopy (MRS), yielding chemical information, is the oldest. The resonance frequency of protons is affected by their chemical environment, such that each compound has its own unique MR spectrum. This allows the detection and quantification of metabolites that are abundantly present in the brain (Oz et al., 2014).

With brain MRS, N-acetylaspartate (NAA, with a singlet resonance at 2.0 ppm and a multiplet around 2.6 ppm), choline-containing compounds (Cho, singlet at 3.2 ppm) and creatine (including phosphocreatine) (Cr, two singlet resonances at 3.0 and 3.9 ppm) are easily identified. NAA is seen as marker of neuronal integrity. Cho is a measure for membrane density and rate of turnover. Although not valid in all pathologies, Cr is often regarded relatively stable and therefore used as reference to calculate metabolite ratios. It can also be regarded a marker of cell density. Other important metabolites are myo-inositol (mI) and lactate (Lac). MI is a glial metabolite and is elevated in astrogliosis. Lac is typically not detectable, but is elevated with anaerobic glycolysis (Barker and Horska, 2004, Oz, G., Alger, J.R., Barker, P.B., Bartha, R., Bizzi, A., Boesch, C., Bolan, P.J., Brindle, K.M., Cudalbu, C., Dincer, A., Dydak, U., Emir, U.E., Frahm, J., Gonzalez, R.G., Gruber, S., Gruetter, R., Gupta, R.K., Heerschap, A., Henning, A., Hetherington, H.P., Howe, F.A., Huppi, P.S., Hurd, R.E., Kantarci, K., Klomp, D.W., Kreis, R., Kruiskamp, M.J., Leach, M.O., Lin, A.P., Luijten, P.R., Marjanska, M., Maudsley, A.A., Meyerhoff, D.J., Mountford, C.E., Nelson, S.J., Pamir, M.N., Pan, J.W., Peet, A.C., Poptani, H., Posse, S., Pouwels, P.J., Ratai, E.M., Ross, B.D., Scheenen, T.W., Schuster, C., Smith, I.C., Soher, B.J., Tkac, I., Vigneron, D.B., Kauppinen, R.A., Group, M.R.S.C., 2014). For a detailed overview, we refer to Oz (Oz et al., 2014).

**Fig. 8:**
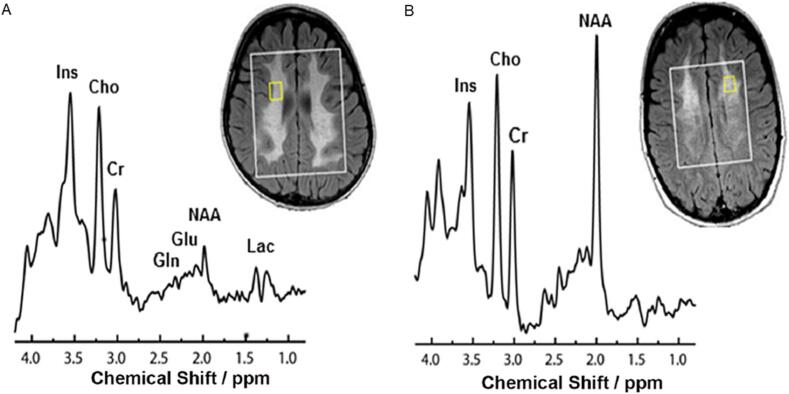
MR spectra of two patients with metachromatic leukodystrophy (MLD). A) Juvenile MLD with a poor outcome. N-acetylaspartate (NAA) concentration is highly decreased, while lactate (Lac) is increased. B) Juvenile MLD with good outcome. NAA concentration is normal and no Lac is detected.

Adapted from van Rappard et al., 2018a

Due to the differences in tissue composition, substantial regional and age-related differences in metabolite concentrations exist in the healthy brain (Barker and Horska, 2004, Kreis et al., 2002, Pouwels et al., 1999). In leukodystrophies, MRS is used extensively and can play a role on various levels (Brockmann et al., 2003a, Brockmann et al., 2003b, Brockmann et al., 1996, Farina et al., 2000, Feldmann et al., 2023, Finnsson et al., 2013, Hanefeld et al., 2005, Martin et al., 2012, Nelson et al., 2013, Pizzini et al., 2003, van Egmond et al., 2013, van Rappard et al., 2018a, Zanigni et al., 2015). The lack, abundance or abnormal presence of a metabolite can be diagnostic for specific leukodystrophies. An example includes the presence of prominent resonances between 3.6 and 3.8 ppm, indicative of arabitol and D-ribitol, diagnostic of ribose-5-phosphate isomerase deficiency (Huck et al., 2004). Canavan disease is characterized by a highly elevated NAA-signal (Austin et al., 1991, Janson et al., 2006). The detection (and thereby elevation) of succinate at 2.4 ppm indicates succinate dehydrogenase deficiency (Helman et al., 2016). Predictions on histopathology are also possible. Cerebral lesions in X-linked adrenoleukodystrophy have multiple zones, which have distinct spectra. In the central, burned-out zone, a strong decrease in NAA is indicative of the axonal loss. The second, inflammatory zone of the lesion is characterized by a strong increase of mI and Lac. The outermost, actively demyelinating zone is characterized by increased Cho, related to enhanced membrane turnover (Eichler et al., 2002, van der Voorn et al., 2011). The detection of MRS abnormalities before the onset of neurological symptoms may help in the selection of patients for treatment with hematopoietic stem cell transplantation (Pouwels et al., 1998). Similarly, in patients with MLD, metabolites at baseline scan may distinguish patients with good outcome after hematopoietic stem cell transplantation from patients with a poor outcome (Fig. 8)(van Rappard et al., 2018a). MRS also differentiates between classes of leukodystrophies, although this depends on the disease stage as well. Cho is for instance often decreased in patients with hypomyelination, while it is elevated in patients with a demyelinating leukodystrophy (Steenweg et al., 2016, van der Voorn et al., 2011).

MRS is associated with various limitations. The largest limitation is the low concentration of metabolites, with an inherently low signal. Thus, the spatial resolution is low and relatively large volumes-of-interest are needed. The determination of absolute metabolite concentrations is challenging, and metabolite ratios are by definition influenced by two metabolites. A multitude of choices regarding acquisition parameters (such as localization method, TR, and TE) and analysis methods hinder comparison between studies. Recently, an international expert panel published recommendations on acquisition parameters and reporting standards for MRS (Kreis, R., Boer, V., Choi, I.Y., Cudalbu, C., de Graaf, R.A., Gasparovic, C., Heerschap, A., Krssak, M., Lanz, B., Maudsley, A.A., Meyerspeer, M., Near, J., Oz, G., Posse, S., Slotboom, J., Terpstra, M., Tkac, I., Wilson, M., Bogner, W., Experts' Working Group on Terminology for, M.R.S., 2020, Lin et al., 2021, Maudsley et al., 2020, Oz, G., Deelchand, D.K., Wijnen, J.P., Mlynarik, V., Xin, L., Mekle, R., Noeske, R., Scheenen, T.W.J., Tkac, I., Experts' Working Group on Advanced Single Voxel, H.M., 2020). Currently, the long acquisition times, sensitivity to field inhomogeneities and extracranial lipid contamination limit the possibility to acquire whole-brain MRS. In the future, spectral resolution may profit from technical improvements currently developed at higher field strength regarding coil design and improved shimming, making whole-brain MRS feasible (Maudsley et al., 2020, Trattnig et al., 2018).

## 5 Challenges and opportunities

Significant progress in quantitative MRI techniques has occurred and their full potential has yet to be determined. Various techniques offer quantitative information on white matter components and together they offer a unique insight into the complexity of the white matter microstructure. There is currently not a single technique that serves as gold standard to describe the white matter microstructure. Techniques are in different stages of development (Granziera et al., 2021). Some techniques are already available on clinical scanners, while other techniques are at the level of research, and need to be implemented on the scanner by dedicated MR physicists.

Most techniques are novel, and it is required to investigate normal variation, before interpretation of results in patients is possible. Currently, such results are more readily available in the adult population (Cox et al., 2016, Kiely et al., 2022). The largest normal change in white matter composition and microstructure is related to myelination, which mainly occurs in the first three years of life, but only reaches completion in young adulthood. Additionally, during childhood and adolescence, axonal packing increases (Lebel and Deoni, 2018). It is, therefore, crucial that for novel methods the evolution of tissue properties during development is known and that (regional) normal values for all ages are collected. With these, it may become clear that an apparent change in microstructural properties on sequential studies in young patients in fact reflects normal development instead of remittance or progression of the disease or a therapeutic effect.

There are other issues that must still be solved. Model-based techniques, including NODDI, all multi-compartment relaxometry methods and SyMRI, depend on fixed assumptions or constraints in their analyses, which may be valid in healthy adult white matter, but which may introduce bias in infants, in gray matter, and in pathologies. Regarding MWF, a priori constraints may prevent visualization of water compartments with changed relaxation times or may not correctly estimate their volume. For example, if myelin sheaths are less compact, the T2 relaxation time of this compartment is prolonged, and MWF based on an upper limit (for instance, 40 ms) would underestimate the myelin content (Odrobina et al., 2005). So, what is measured as “MWF” in pathological tissue has to be interpreted with care, and cannot be regarded straightforwardly as representative of the myelin content. Another example concerns NODDI, which is optimized for normal adult white matter; it has become clear that the intrinsic parallel diffusivity of neurites, one of the default fitting variables, offers a suboptimal reflection of the microstructural properties of the white matter of infants and of gray matter (Guerrero et al., 2019). Thus, assumptions based on healthy adult white matter may lead to biased results in structurally or chemically different tissue (Piredda et al., 2020). Importantly, however, the underlying relaxation times and diffusion metrics derived with the applied sequences are determined without constraints, and still provide additional useful information.

Since the microstructure models are complex, a combination of techniques may help to improve insight into brain tissue microstructure. An example is MCR-DIMWI, which utilizes information from multi-shell DWI, both the volume fractions of the compartments and the frequency shifts from the white matter fiber orientations, to make fitting of T1 and T2* multi-compartment relaxometry more robust (Chan and Marques, 2020).

Another interesting possibility is utilizing different techniques to generate new microstructural results. In histopathology, the g-ratio is defined as the ratio of the inner axonal diameter to the total outer diameter, comprising axon and myelin, which is a measure of the myelin sheath thickness relative to axon size (Chomiak and Hu, 2009). By combining methods estimating myelin volume fraction and methods estimating axonal volume fraction, the g-ratio within a voxel can be calculated (Ellerbrock and Mohammadi, 2018, Jung et al., 2018, Stikov et al., 2015).

The interpretation of results from quantitative MR would greatly benefit from direct correlation with histopathology. Unfortunately, especially for novel techniques such studies are scarce and they have been performed in animals or post mortem human brain tissue, with effects of post-mortem delay and potentially formalin fixation (Chan et al., 2022). Two *meta*-analyses, which included a total of 69 studies comparing any quantitative MRI measure to a histology measure, showed that the correlation coefficient between quantitative MR and histology metrics varied strongly, not only between different MR techniques, but also within the same MR technique (Lazari and Lipp, 2021, Mancini et al., 2020, van der Weijden et al., 2021). In the absence of the ability to make direct MR-histopathology correlations, an alternative option is to validate a new technique with a well-known existing technique. For instance, ihMT results correlate much stronger with 3D GRASE-derived MWF than MTR, suggesting that ihMT is more specific for myelin than MTR (Ercan et al., 2018, Vavasour et al., 1998). Of course, this does not replace the need for a true gold standard.

Reproducibility, reliability and comparability of the results of the different quantitative techniques are of concern. Reproducibility is typically expressed as coefficient of variation (COV), which is the standard deviation divided by the sample mean. For quantitative parameters whole brain white matter COVs can be as low as 2–10%, whereas smaller ROIs typically have higher COVs (Andica et al., 2020, Gracien et al., 2020, Lee et al., 2018, Lehmann et al., 2021, Meyers et al., 2013, Oh et al., 2006). In general, field inhomogeneities lead to higher COVs, underscoring the need for B1-inhomogeneity correction. This has been observed for ROIs in frontal white matter, but can also be expected for white matter in other lobes (Lee et al., 2018). Reproducibility is better in a single-site, single-scanner setting than in a multi-site, multi-vendor setting (Andica et al., 2020, Meyers et al., 2013). With increasing field strengths, relaxation times change and MWF values are several percentage-points higher, hindering comparison of studies performed at different field strengths (Labadie et al., 2014, Oh et al., 2006).

Most quantitative MRI techniques focus on a subset of microstructural white matter components. Myelin- and axon-related metrics are best represented by the available techniques. However, for disease monitoring, all white matter components should be considered. Astrocytes and microglia are currently underexposed in neuroimaging, while their importance is indisputable (Vainchtein and Molofsky, 2020). Exploratory steps are taken to also extract information about these cells by quantifying microglia and astrocyte activation within gray matter in humans in vivo using multi-shell DWI (Garcia-Hernandez et al., 2021).

Biomarkers may be useful in various stages of disease: in making a diagnosis, assessing disease severity, estimating disease prognosis and monitoring (Atkinson et al., 2001). For an imaging parameter to become a relevant biomarker, several requirements must be met. The measure needs to be robust, quantitative, reproducible, and sensitive to change. Most importantly, clinical meaningfulness must be demonstrated in the disease of interest by showing that variation in the parameter relates to variation in the clinical disease course (Atkinson et al., 2001). Both the accuracy (the correlation of the measure with the clinical endpoint) and precision (the reproducibility of the measure) must be demonstrated (Atkinson et al., 2001).

For monitoring white matter diseases, a scanning protocol of multiple techniques giving complementary information should exist. Qualitative assessment of white matter and visual rating scores can be obtained with conventional MRI, such as T2-weighted, T1-weighted and FLAIR imaging. To aid segmentation and volumetry, isometric 3D T1-weighted and FLAIR images covering the entire brain are useful. They can be obtained with any clinical MR machine. To allow more detailed quantitative monitoring of microstructural white matter components, other techniques can be added. Myelin content can be estimated by calculating the MWF using one of the multi-component relaxometry techniques. Neurite density and orientation dispersion, and water content can be estimated by modeling multi-shell DWI data. Furthermore, a single shell from the multi-shell DWI provides commonly used DTI-metrics, like FA, overall diffusivity and axial versus radial diffusivity. The chemical information provided by MRS is relevant to assess membrane content and turnover, and axonal health. The local leukodystrophy scanning protocol should be tailored to the possibilities of the MR machine and the expertise of the local center, taking time constraints into account. Using one protocol for all leukodystrophies not only allows longitudinal monitoring, but also comparing pathology in different disorders.

With all the different possibilities of MRI, it is a challenge to keep the total scan time clinically feasible. Besides critical appraisal on which techniques are included in a protocol, a decrease in acquisition time per sequence is valuable. Acquisition can be accelerated using methods such as parallel imaging, compressed sensing, partial Fourier, and lowering spatial resolution (Kozak et al., 2020). Post-processing solutions that use artificial intelligence to optimize the data acquired are also being investigated (Kozak et al., 2020).

## 6 Conclusion

Quantitative MRI techniques allow obtaining microstructural information in a non-invasive fashion and nowadays have a reasonable acquisition time. Choosing multiple quantitative techniques within a scanning protocol is preferred, as they offer complementary information. Total scanning time remains an important factor to consider when deciding on the scanning protocol. The whole-brain multi-shell DWI and myelin water imaging techniques are particularly advanced in their development and therefore commendable, although their correlations with clinical disease and histopathology are to be further explored. We expect that quantitative MR techniques have the potential to serve as secondary outcome measures in treatment trials of leukodystrophies.

## Declaration of Competing Interest

The authors declare that they have no known competing financial interests or personal relationships that could have appeared to influence the work reported in this paper.

## Data Availability

No data was used for the research described in the article.
